# Diversity of Multicellular Magnetotactic Prokaryotes in Sanya Haitang Bay

**DOI:** 10.3390/microorganisms13112624

**Published:** 2025-11-19

**Authors:** Jiangxue Shi, Wenyan Zhang, Yi Dong, Yao Liu, Min Liu, Tian Xiao, Long-Fei Wu, Hongmiao Pan

**Affiliations:** 1 CAS Laboratory of Marine Ecology and Environmental Sciences, Institute of Oceanology, Chinese Academy of Sciences, Qingdao 266071, China; shijx@qdio.ac.cn (J.S.); zhangwy@qdio.ac.cn (W.Z.); yidong@qdio.ac.cn (Y.D.); txiao@qdio.ac.cn (T.X.); 2 Laboratory for Marine Ecology and Environmental Sciences, Qingdao Marine Science and Technology Center, Qingdao 266237, China; 3 University of Chinese Academy of Sciences, Beijing 100049, China; 4 Sino-French Joint Laboratory for Evolution and Development of Magnetotactic Multicellular Prokaryotes (LIA-MagMC), Qingdao 266071, China; wu@imm.cnrs.fr; 5 The Public Technology Center of the Institute of Oceanology, Chinese Academy of Sciences, Qingdao 266071, China; liuyao@qdio.ac.cn; 6 School of Ecology and Environment, Hainan Tropical Ocean University, Sanya 572022, China; minliu@hntou.edu.cn; 7 CNRS, Laboratory of Bioenergetics and Cell Biology (LCB), Aix-Marseille University, 31 Chemin Joseph Aiguier, 13402 Marseille, France

**Keywords:** multicellular magnetotactic prokaryotes, intertidal zone, diversity, magnetosomes, phylogenetics

## Abstract

The intertidal sediments of Sanya Haitang Bay, a tropical coast, harbor abundant multicellular magnetotactic prokaryotes (MMPs). Using light and electron microscopy, micromanipulation sorting, and whole-genome amplification, we examined their diversity from morphological, phylogenetic, and ecological perspectives. Two types of MMPs were identified: ellipsoidal (eMMPs) and spherical (sMMPs). Their average abundance was 1.37 × 10^3^ ind./dm^3^ in autumn and 0.27 × 10^3^ ind./dm^3^ in spring, indicating strong seasonal variation. eMMPs averaged 9.74 × 8.15 µm, consisting of 80–100 cells arranged in layers, whereas sMMPs averaged 5.64 µm in diameter with 40–50 cells organized radially or spirally. Electron microscopy revealed bullet-shaped magnetosomes in both types: those in eMMPs averaged 90.1 × 34.0 nm, while those in sMMPs averaged 97.2 × 36.3 nm. Interestingly, Cu was homogenously detected in the magnetosomes of sMMPs. 16S rRNA gene analysis identified nine OTUs, including three potential new species in the Desulfobacteraceae family within Thermodesulfobacteriota phylum. Of these, two may represent a new genus, and one is affiliated with *Candidatus* Magnetananas. Global distribution analysis suggests that eMMPs prefer stable, nutrient-rich environments, whereas sMMPs occupy broader ecological niches. Together, these findings expand understanding of tropical MMP diversity and distribution, and the discovery of Cu-containing magnetosomes provides new insight into biomineralization mechanisms.

## 1. Introduction

Magnetotactic bacteria (MTB) are a group of Gram-negative bacteria with a unique magnetosensing capability, enabling them to perceive and orient along Earth’s magnetic field lines. They were first discovered by Bellini in 1963 [1], but it was not until Blakemore’s research was published in Science in 1975 [2] that this field gradually became a hotspot in microbiological and ecological research. According to the Genome Taxonomy Database (GTDB), MTB now span up to 17 phyla, primarily distributed within the Pseudomonadota phylum, as well as the Desulfobacterota, Nitrospirota, Omnitrophota, Planctomycetota, and Latescibacteria phyla [3]. Morphologically, MTB are mainly divided into two types: unicellular and multicellular aggregates. Unicellular MTB exhibit diverse morphologies, commonly including coccoid, spirillum, rod and vibrioid forms [4,5,6,7,8,9]. In contrast, multicellular aggregates, known as multicellular magnetotactic prokaryotes (MMPs), represent a unique morphological type that maintains a multicellular organization throughout their life cycle. In 1983, they were first discovered and reported by Farina in the Freitas Lagoon of Rio de Janeiro, Brazil [10].

Based on 16S rRNA gene sequences, MMPs are classified within the family Desulfobacteraceae of the phylum Thermodesulfobacteriota (formerly class Deltaproteobacteria of the phylum Proteobacteria). Morphologically, MMPs are generally categorized into two main types according to their shape and cellular arrangement: ellipsoidal MMPs (eMMPs) with a pineapple-like organization, and spherical MMPs (sMMPs) with a rosette-like (or mulberry-like) organization [11,12]. Typically, eMMPs range from 8 to 23 μm in length and 7 to 17 μm in width [13]. It comprises approximately 28–101 cells arranged in stratified, interlocking layers around a central cavity, forming a centrosymmetric structure [12,14,15,16]. In contrast, sMMPs are 3–12 μm in diameter and consist of 10–40 cells arranged spirally around an acellular internal cavity, yielding a radial architecture [15,17,18,19,20].

With the exception of unique non-magnetosome-forming MMPs (nMMPs) reported from low-salinity non-marine habitats in the United States [21], all confirmed MMPs have been found in marine habitats. They are able to synthesis nanoscale magnetosome crystals within their cells, which are primarily bullet-shaped Fe_3_O_4_ and/or irregularly shaped Fe_3_S_4_ [20,22,23]. A notable exception is the Wadden Sea isolate *Candidatus* Magnetomorum litorale, an sMMP that synthesizes bullet-shaped Fe_3_S_4_ magnetosomes [24]. MMPs display peritrichous flagellation and exhibit several sophisticated motility behaviors, including magneto-aerotaxis [4,25], escape motility (ping-pong motility) [16,26,27,28,29], and photophobic responses [18,30,31,32,33]. These behaviors not only demonstrate the adaptive capacity of MMPs in complex environments but also provide important insights into their ecological distribution and survival strategies.

Currently, 88 full-length 16S rRNA gene sequences of MMPs have been deposited in the NCBI database, demonstrating a broad global distribution across diverse habitats in South America, North America, Europe, and Asia. These include Brazilian lagoons, hypersaline lakes in the United States, intertidal sediments in Marseille (France), sandy coastal areas of the Wadden Sea (Germany), as well as Chinese sites such as the Yuehu Lake intertidal zone in Rongcheng, Qingdao intertidal zones, Sanya mangrove forests, and coral reefs in the South China Sea [14,15,16,17,24,31,34,35,36,37,38,39,40]. Different habitats possess unique physical, chemical, and biological characteristics, such as salinity variations, chemical gradients, sediment properties, and biological community structures. These conditions provide diverse ecological niches for MMPs and thereby shape their remarkable diversity. As a result, MMPs exhibit significant ecological adaptability and evolutionary importance within complex aquatic ecosystems.

This study focuses on MMPs in the intertidal zone of Haitang Bay, Sanya, which represents the southernmost known sampling site of MMPs in the Northern Hemisphere. By integrating microscopy, electron microscopy, micromanipulation-based sorting, and whole-genome amplification (WGA) techniques, we investigated the morphological traits and taxonomic diversity of MMPs from distributional, morphological, and phylogenetic perspectives. The findings provide new evidence supporting the widespread distribution of MMPs in intertidal environments and suggest their crucial ecological functions within these habitats.

## 2. Materials and Methods

### 2.1. Sample Collection, Enrichment, and Observation

**Figure 1:**
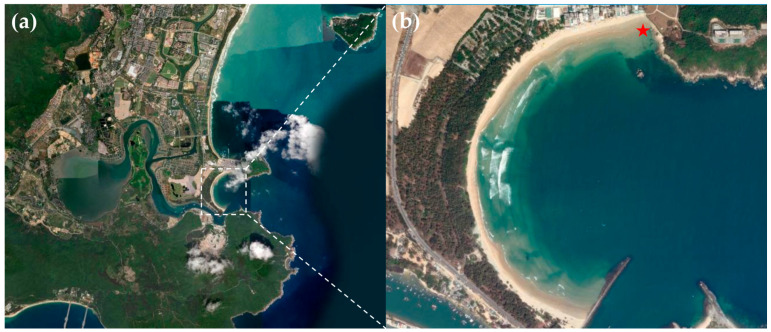
Sampling Site in Haitang Bay, Sanya. (**b**) is an enlarged view of the area within the dashed box in (**a**), with the red “☆” indicating the specific sampling point. The map is sourced from: https://www.arcgis.com (accessed on 4 July 2025).

Haitang Bay is located on the northeastern coast of Sanya City, Hainan Province, China (Figure 1a). Its unique semi-circular, pocket-like topography generates diverse and variable wave patterns at different sites, increasing the complexity of the local marine environment. Furthermore, the bay is known for its rich coral diversity. Studies indicate that the average coral coverage in Houhai Bay reaches 53.83%, with a high recruitment rate (4.5 ind.·m^−2^), suggesting considerable self-recovery capacity [41]. In addition, areas such as Wuzhizhou Island host up to 137 recorded coral species [42]. A seagrass bed dominated by *Thalassia hemprichii* and *Cymodocea rotundata*, covering approximately 0.06 km^2^, also extends from the Wuzhizhou Island freight dock to rocky reef zones [43].This distinctive geomorphology and biodiversity, under the influence of tidal movements and water flow fluctuations, promote the transport of significant nutrients into the bay. These conditions support a productive and highly diverse marine ecosystem, making Haitang Bay an important area for marine biological resources and a key site for ecological research. In this study, sampling was conducted at a site in Haitang Bay (109.726776° E, 18.272547° N), indicated by a red star in Figure 1b.

Surface sediments (0–10 cm depth) were collected from the intertidal zone of Haitang Bay on 12 September 2024, and 27 March 2025, with 34 and 54 samples obtained on each date, respectively. Each sample was stored in a 500 mL bottle and supplemented with in situ seawater at a 1:1 ratio (sediment: seawater). All bottles were transported to the laboratory and stored statically in the dark for 1–2 weeks. Prior to enrichment, we thoroughly mixed the seawater and sediment in each bottle. A bar magnet (field strength approximately 0.05 T) was then fixed 1 cm above the sediment-seawater interface with its S pole facing the bottle wall. Following 30 min of magnetic enrichment in the dark, we collected material accumulated near the magnet using a Pasteur pipette and transferred it to a 1.5 mL centrifuge tube (recorded as volume V_1_). This enriched sample was gently mixed by inversion, and a 20 μL (V_2_) aliquot was placed on a glass slide. MMPs were then counted in triplicate under an optical microscope (Olympus BX51, Tokyo, Japan) using the “hanging drop method” [44] under an applied magnetic field. The average count (N) from the three replicates represented the absolute number of MMPs in the bottle.

MMP abundance (A, in ind.·dm^−3^) was calculated as follows: A = N × V_1_/(V_2_ × V) × 1000 (N is the average MMP count from triplicate measurements (ind.); V_1_ is the volume of the enriched sample transferred to the centrifuge tube (mL); V_2_ is the volume of the aliquot used for counting (μL); V is the volume of the original sediment sample (cm^3^); The factor 1000 converts V_2_ from μL to mL for unit consistency.) We observed and photographed MMP morphology and motility behavior. Cell dimensions were measured using ImageJ software (version 1.53C).

### 2.2. Morphological Analysis of Multicellular Magnetotactic Prokaryotes

#### 2.2.1. Scanning Electron Microscopy (SEM)

Purified MMPs obtained via magnetic collection were further refined using the racetrack method [45]. The samples were fixed with 1.25% glutaraldehyde at 4 °C for 12 h. Fixed cells were vacuum-filtered onto a 2 μm pore-size polycarbonate membrane using a diaphragm vacuum pump (GM-0.33, Tianjin, China) and gently rinsed twice with phosphate-buffered saline (PBS) to remove residual fixative. Subsequently, the samples were dehydrated through a graded ethanol series (50%, 60%, 70%, 80%, 90%, and 100%; 10 min per step), followed by substitution with isoamyl acetate. Critical point drying was performed to preserve structural integrity, and the specimens were sputter-coated with gold. Morphological observations were carried out using a cold-field emission scanning electron microscope (SEM; Zeiss Gemini 500, Oberkochen, Germany).

#### 2.2.2. Transmission Electron Microscopy (TEM)

Three microliters aliquot of purified MMPs was dropped to a TEM copper grid. When the liquid had nearly evaporated, the grid was rinsed twice with 5 μL of ultrapure water. Excess liquid was carefully removed, and the dried grid was stored in a grid box within a desiccator.

The samples were subsequently observed using two instruments: a 120 kV transmission electron microscope (TEM; Hitachi HT7700, Tokyo, Japan) and a 200 kV field-emission transmission electron microscope (TEM; JEM-F200, Tokyo, Japan). These were employed to analyze cellular dimensions, as well as the morphology, arrangement, and distribution of magnetosomes. High-resolution TEM (HRTEM) imaging was performed to obtain lattice fringes of magnetosome crystals. Elemental composition was assessed using energy-dispersive X-ray spectroscopy (EDXS) elemental mapping and point spectroscopy. Phase identification was carried out by comparing X-ray diffraction (XRD) patterns, acquired through integrated analytical capabilities, with standard reference cards using Jade software (version 6.0). Magnetosome crystals were counted and measured using ImageJ software.

##### 2.3. 16S rRNA Gene Sequence Analysis of MulticellularMagnetotactic Prokaryotes

Individual MMPs of distinct morphotypes were isolated under an inverted microscope (Olympus IX51, Tokyo, Japan) equipped with a TransferMan ONM-2D micromanipulator and an IM-9B CellTram Oil manual hydraulic pressure control system, (Narishige, Tokyo, Japan) [14,19,20]. Using a glass capillary needle filled with mineral oil, target cells were aspirated and sequentially transferred through droplets of freshly filtered seawater to remove environmental contaminants. This process was repeated until the transferred sample contained exclusively MMPs, as visually confirmed. The purified cells were finally expelled into PBS buffer and stored at −80 °C.

Whole-genome amplification (WGA) of individually sorted MMPs was performed via multiple displacement amplification (MDA) using the REPLI-g Single Cell Kit (Qiagen, Germany) according to the manufacturer’s instructions. The amplified products were used as templates to amplify the near-full-length 16S rRNA gene with primers 27F (5′-AGAGTTTGATCCTGGCTCAG-3′) and 1492R(5′-GGTTACCTTGTTACGACTT-3′) [46]. The resulting PCR amplicons were electrophoresed on a 1% agarose gel, purified, and cloned into the pMD18-T vector (Takara, Kusatsu, Japan). The ligated products were transformed into *E. coli* DH5α (Takara, Japan) competent cells. Positive clones were randomly selected, and Sanger sequencing of the inserted 16S rRNA gene was performed by Shanghai Bioengineering Co., Ltd. (Shanghai, China).

After obtaining the clone sequences, BLAST comparisons were performed against the NCBI database. Only those sequences showing the highest similarity to known MMP 16S rRNA genes were retained for further analysis. These sequences were trimmed and aligned using BioEdit (version 7.2.5). Operational Taxonomic Units (OTUs) were defined at a ≥99% similarity threshold [47]. Representative sequences from each OTU were compared against the nr/nt database using the NCBI BLAST online tool (https://blast.ncbi.nlm.nih.gov/Blast.cgi, accessed on 2 April 2025). All MMP-derived OTU sequences and their closest reference matches were aligned using Clustal W in IQ-TREE, manually refined, and used to construct a phylogenetic tree via the Neighbor-Joining method with 1000 bootstrap replicates. The resulting tree was visualized and refined using the iTOL online platform (https://itol.embl.de, accessed on 2 April 2025). The 9 OTU sequences obtained in this study have been deposited in the GenBank database under accession numbers PV810351–PV810359.

## 3. Results

### 3.1. Abundance and Motility Behaviors of Multicellular Magnetotactic Prokaryotes

MMPs collected from sediments containing coral debris (Figure S1) were observed and statistically analyzed using optical microscopy. Among the 34 samples obtained on 12 September 2024 (autumn), MMPs were detected in approximately 76.47% (26/34) of the samples, with an average abundance of 1.37 × 10^3^ ind./dm^3^. Among MMP-positive samples, the majority (46.15%, 12/26) contained both eMMPs and sMMPs morphotypes. Samples containing only sMMPs accounted for 38.46% (10/26), while those with only eMMPs constituted 15.38% (4/26).

In samples collected on 27 March 2025 (spring), MMPs were present in approximately 62.96% (34/54) of the samples, with an average abundance of 0.27 × 10^3^ ind./dm^3^. Within these, 17.65% (6/34) contained both morphotypes, while samples with only sMMPs accounted for 67.65% (23/34), and those with only eMMPs represented 14.71% (5/34). These results suggest seasonal variations in the distribution and composition of MMPs.

Furthermore, during microscopic observation, MMPs exhibited characteristic escape motility (also referred to as “ping-pong” motion). Both eMMPs and sMMPs displayed rotational movement along their major structural axes—the long axis for eMMPs and the central axis for sMMPs. Additionally, a “random walk” behavior was observed along the edge of droplets in applied magnetic field (Video S1).

### 3.2. Morphology of Multicellular Magnetotactic Prokaryotes

**Figure 2:**
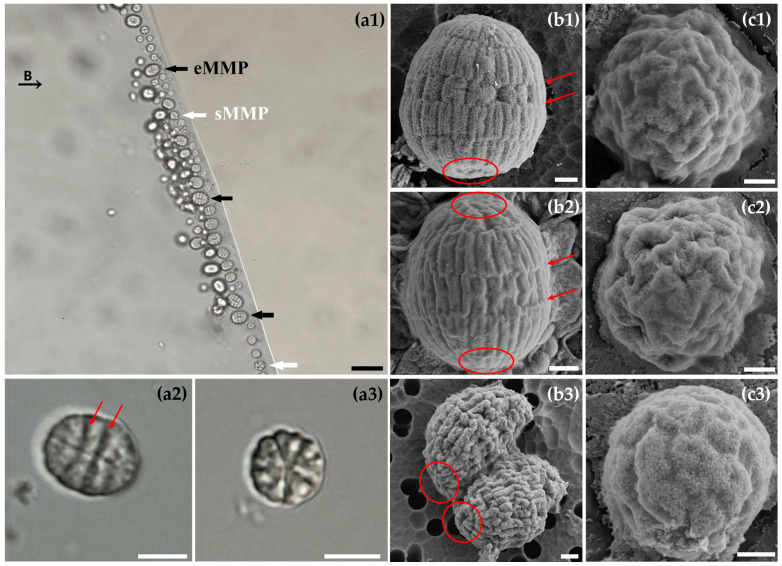
The morphology of MMPs. Light microscopic images of eMMPs and sMMPs (the letter “B” and the thin black arrow indicate the direction of the external magnetic field, Under an external magnetic field that MMPs aggregate at the edge of the droplet with black thick arrows indicating eMMPs and white thick arrows indicating sMMPs) (**a1**), eMMPs magnified 100× under light microscope (**a2**), sMMPs magnified 100× under light microscope (**a3**). Morphology of eMMPs under SEM (The red arrows in **b1** and**b2** correspond to the grooves between layers of eMMPs indicated by the red arrows in **a2** under the light microscope, which are aligned parallel to the short axis of the ellipsoid. The red circles in **b1** and **b2** mark the slightly invaginated basal cells of the sixth layer and the outward protrusions at both poles of the eMMP, respectively. The red circle in **b3** marks the basal structures of two prospective offspring at the end of the eMMP undergoing fission.) (**b1**–**b3**). Morphology of sMMPs under SEM (**c1**–**c3**). (Scale bars: (**a1**): 20 μm; (**a2**,**a3**): 5 μm; (**b1**–**b3**): 1 μm; (**c1**–**c3**): 500 nm).

Both eMMPs and sMMPs morphotypes were observed under light microscopy (Figure 2a1–a3). The eMMPs measured approximately 9.74 ± 1.52 µm in length and 8.15 ± 1.54 µm in width (*n* = 40) (Figure 2a1, black arrow) and consisted of 5–7 concentric cell layers. Distinct grooves between layers, aligned parallel to the short axis of the ellipsoid, were clearly visible (Figure 2a2, arrow).

SEM revealed the detailed architecture of intact eMMPs (Figure 2b1,b2). The cells were arranged in multiple stratified layers. Cells in the middle layers predominantly exhibited an “H” shape, while those at the apical and basal poles displayed an “A” shape, converging at their apexes. Adjacent layers interlocked via “H–H” or “H–A” connections, forming a tightly enclosed ellipsoidal structure. These intercellular junctions corresponded to the grooves observed under light microscopy (Figure 2a2,b1,b2, red arrows). When stratified along the long axis, with the apical most cells designated as Layer 1, the eMMP in Figure 2b1 consisted of 6 layers and approximately 96 cells. Layer 1 (A-shaped cells) had a base width of 0.73 ± 0.20 µm (*n* = 4) and a height of 1.37 ± 0.18 µm (*n* = 4). Layers 2, 4, and 5 comprised elongated H-shaped cells with a height of 1.54 ± 0.10 µm (*n* = 13) and a width of 0.63 ± 0.11 µm (*n* = 13). In contrast, Layer 3 consisted of shorter, broader H-shaped cells (height: 0.97 ± 0.12 µm, *n* = 8; width: 1.01 ± 0.18 µm, *n* = 4) with conspicuous gaps between them. The basal cells of the sixth layer appear to be slightly invaginated toward the central cavity (Figure 2b1, red circle).

Another eMMP, shown in Figure 2b2, consisted of 5 layers and approximately 78 cells. The apical (Layer 1) and basal (Layer 5) layers contained A-shaped cells with a base width of 0.85 ± 0.07 µm (*n* = 3) and a height of 1.28 ± 0.11 µm (*n* = 3). Layers 2–4 were composed of H-shaped cells. Notably, Layer 3 again featured stout H-shaped cells (height: 1.34 ± 0.24 µm, *n* = 7; width: 0.82 ± 0.05 µm, *n* = 4), while the other layers contained slender H-shaped cells (height: 1.39 ± 0.13 µm, *n* = 14; width: 0.48 ± 0.12 µm, *n* = 14). The overall structure showed outward protrusions at both poles (Figure 2b2, red circle). These findings indicate that eMMPs exhibit pronounced cellular morphological differentiation, consistent with our previous observations [14].

Furthermore, an eMMP with a morphology suggestive of fission was observed via SEM (Figure 2b3). Division initiated from one pole of the long axis, consistent with previous reports [12]. The undivided end had already developed the basal structures of two prospective offspring (Figure 2b3, red circle), providing further evidence that MMP reproduction occurs without a dissociative unicellular stage. It is hypothesized that a multicellular individual first increases its cell number until two connected multicellular units are formed, followed by fission along the long axis to yield two separate multicellular progenies.

The sMMPs had an average diameter of 5.64 ± 0.80 µm (*n* = 8; Figure 2a1, white arrow) and exhibited a radial, rosette-like symmetry under light microscopy (Figure 2a3). SEM revealed variations in their cellular organization. Some sMMPs consisted of 38–40 twisted cells arranged in a radial mosaic pattern (Figure 2c1,c2), while others contained approximately 46 circular or near-ellipsoidal cells organized spirally (Figure 2c3).

### 3.3. Magnetosome Characteristics of Multicellular Magnetotactic Prokaryotes

**Figure 3:**
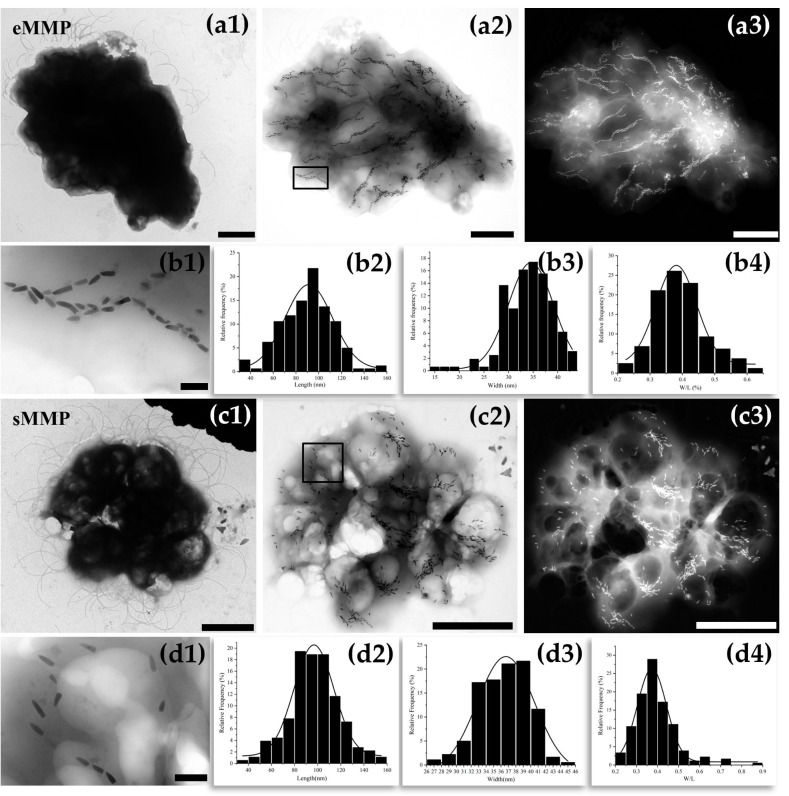
The arrangement and size of the magnetosomes in eMMP and sMMP. Images of eMMP taken under TEM (**a1**) STEM bright-field (**a2**) and dark-field conditions (**a3**). Magnified view of the magnetosomes in eMMP cells indicated by the black box in **a2** (**b1**), Statistical distributions of the length (**b2**), width (**b3**) and width-to-length ratio (**b4**) of magnetosomes in eMMP cells. Images of sMMP taken under TEM (**c1**), STEM bright-field (**c2**) and dark-field conditions (**c3**). Magnified view of the magnetosomes in sMMP cells indicated by the black box in **c2** (**d1**), Statistical distributions of the length (**d2**), width (**d3**) and width-to-length ratio (**d4**) of magnetosomes in sMMP cells. (Scale bars: (**a1**–**a3**,**c1**–**c3**): 2.0 μm, (**b1**,**d1**): 200 nm; (**a1**,**c1**) were captured using a 120 kV TEM, while (**a2**,**a3**,**c2**,**c3**) were captured using a 200 kV field-emission TEM).

Based on their distinct projected morphologies (ellipsoidal vs. spherical) and sizes on TEM grids, the observed MMPs were unambiguously classified as either eMMPs or sMMPs. TEM imaging revealed peritrichous flagella on the surface of eMMP cells (Figure 3a1). Under bright-field and dark-field imaging (Figure 3a2,a3), bullet-shaped magnetosomes within eMMP cells were predominantly organized in chains with consistent crystal orientation. In addition to chain formation, irregular clusters of magnetosomes were also observed. The total number of magnetosomes per eMMP was approximately more than 2000. Statistical analysis indicated that the bullet-shaped magnetosomes had an average length of 90.1 ± 22.8 nm, width of 34.0 ± 4.9 nm, and a shape factor (width/length) of 0.39 ± 0.08 (*n* = 166) (Figure 3b1–b4).

sMMP cells also possessed peritrichous flagella, as observed by TEM. (Figure 3c1). Within sMMPs, most bullet-shaped magnetosomes were arranged in clusters, preferentially distributed near the cell periphery, while a minority were dispersed throughout the cytoplasm (Figure 3c2,c3). The total magnetosome count per sMMP was approximately 928. These crystals had an average length of 97.2 ± 21.4 nm, width of 36.3 ± 3.4 nm, and a shape factor of 0.39 ± 0.10 (*n* = 180) (Figure 3d1–d4).

**Figure 4:**
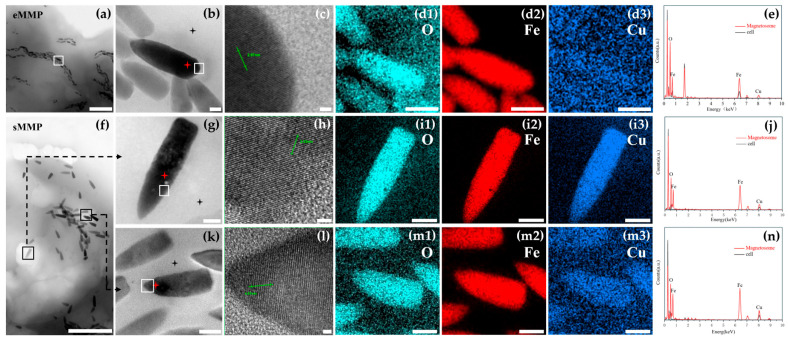
Analysis of composition of magnetosomes within eMMP and sMMP. Analysis of magnetosome composition in eMMP cells by STEM images (**a**) and magnetosomes in eMMP cells is magnified from the white box in a (**b**), HRTEM image of the white-boxed area in **b** (**c**), The EDXS elemental mapping of O, Fe, and Cu in the magnetosomes in **b** (**d1**–**d3**), Spectra of magnetosomes and cytoplasm indicated by red and black stars in **b** (**e**). Analysis of magnetosome composition in sMMP cells by STEM images (**f**) and magnetosomes in sMMP cells is magnified from the black box in f (**g**,**k**), HRTEM images of the white-boxed areas in **g** and **k**, respectively (**h**,**l**), The EDXS elemental mapping of O, Fe, and Cu in the magnetosomes in **g** and **k**, respectively (**i1**–**i3**,**m1**–**m3**), Spectra of magnetosomes and cytoplasm indicated by red and black stars in **g** and **k**, respectively (**g**,**n**). (Scale bars: (**a**,**f**): 500 nm; (**b**,**g**,**k**): 20 nm; (**c**,**h**,**l**): 2 nm; (**d1**–**d3**): 50 nm; (**i1**–**i3**,**m1**–**m3**): 30 nm; The green lines in (**c**,**h**,**l**) represent the measurements of ten lattice spacings in magnetosome crystals, 1 nm = 10 Å).

For further crystallographic analysis, bullet-shaped magnetosomes from eMMPs were examined (Figure 4a,b). HRTEM revealed lattice fringes with a spacing of d = 2.97 Å (Figure 4c), corresponding to the (220) plane of magnetite (Fe_3_O_4_), as confirmed by comparison with the standard XRD pattern (JCPDS card no. 19-0629). EDXS elemental mapping and spectroscopy confirmed that the primary components were iron and oxygen (Figure 4d1–d3,e).

Two bullet-shaped magnetosomes from sMMPs were selected for detailed analysis (Figure 4f,g,k). HRTEM of the crystal in Figure 4g showed well-defined lattice fringes with a spacing of d = 2.53 Å (Figure 4h), matching the (311) plane of magnetite (Fe_3_O_4_). EDXS mapping revealed aggregation of O, Fe, and Cu elements within the magnetosome (Figure 4i1–i3), and EDXS confirmed the presence of Cu in addition to Fe and O (Figure 4j), indicating the incorporation of Cu into the magnetite structure. The magnetosome shown in Figure 4k exhibited a lattice spacing of d = 4.51 Å, which does not correspond to any known crystallographic plane of stoichiometric magnetite. EDXS elemental mapping and spectroscopy consistently detected O, Fe, and Cu in this crystal (Figure 4m1–m3,n), suggesting a possible atypical or doped magnetite phase.

### 3.4. Phylogenetic Analysis of 16S rRNA Genes in Multicellular Magnetotactic Prokaryotes

**Figure 5:**
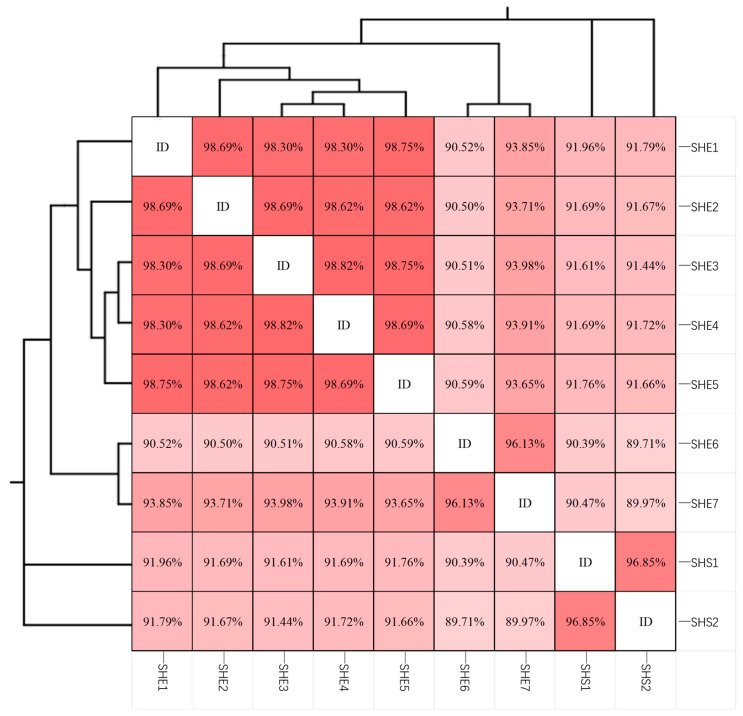
The matrix of MMPs 16S rRNA gene sequence similarity (The higher the similarity, the darker the color).

A total of 120 randomly chosen clones were sequenced. After BLAST comparison in NCBI, 36 sequences were found to be most closely related to known MMP sequences. These sequences were clustered into 9 OTUs at a ≥99% similarity threshold. A similarity matrix (Figure 5) revealed that these OTUs formed three major clades. The first clade comprised eMMPs, consisting of 7 OTUs further subdivided into two subclades. Using sequence similarity thresholds of 97% and 95% for species- and genus-level delineation, respectively [48], the first subclade (SHE1–SHE5) exhibited intra-group similarities between 98.30% and 98.82%, suggesting they belong to the same species. The second subclade (SHE6–SHE7) showed 96.13% similarity, indicating they may represent different species within the same genus. The second and third clades consisted of sMMPs. The similarity between these two clades was 96.85%, suggesting that SHS1 and SHS2 likely represent distinct species within the same genus. Overall, these results indicate that 5 species of MMPs distributed across 3 genera are present in Haitang Bay, Sanya.

The representative sequence of each OTU was aligned with its closest match in the GenBank database (Table 1). Among the 9 OTUs, 7 (SHE1–SHE7) were affiliated with eMMPs and 2 (SHS1, SHS2) with sMMPs. Five OTUs (SHE1–SHE5) showed >97% similarity to known sequences in the database, suggesting they represent known species; these were the dominant taxa, comprising 75.00% (27/36) of the sequences. In contrast, SHE6 and SHE7 exhibited less than 95% similarity to any known MMP 16S rRNA gene sequences. SHE6 (1 sequence) showed highest similarity (94.50%) to an uncultured eMMP prokaryote clone R3–34 (MH013388) from Mediterranean sediments. SHE7 (1 sequence) was most similar (94.03%) to *Candidatus* Magnetananas sp. SF-1 (KT722334) from Xisha coral reefs. The similarity between SHE6 and SHE7 was 96.13% (Figure 5), indicating they may represent two novel species within a new genus. SHS1 (2 sequences) and SHS2 (5 sequences) were most similar to an uncultured delta proteobacterium clone MMP PI7B-6 (KY921895) from Xisha coral reef sediments in the South China Sea, with similarities of 96.99% and 99.54%, respectively. This suggests that SHS2 likely belongs to the same species as clone MMP PI7B-6, while SHS1 may represent a novel species. Therefore, the five species of MMPs identified in the Haitang Bay, which are distributed across three genera, may include two novel species (SHE6 and SHE7) belonging to a new genus, as well as one known species (SHE1–SHE5), one unidentified species (SHS2), and one novel species (SHS1) within two previously described genera.

**Figure 6:**
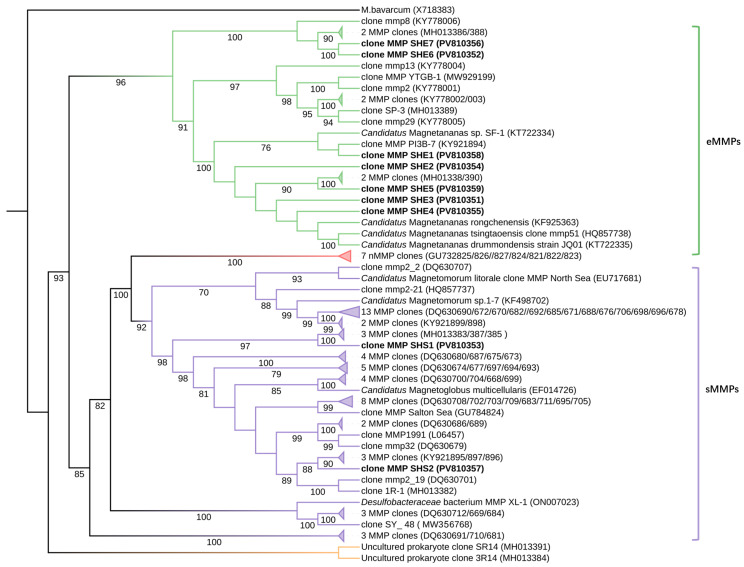
Phylogenetic tree of multicellular magnetotactic prokaryotes (MMPs) based on 16S rRNA gene sequences. The bold sequences represent the 9 OTU sequences of MMPs obtained from this experiment. With *Candidatus* Magnetobacterium bavaricum (X71838) designated as the outgroup, the analysis included a total of 97 MMP taxa (including 88 known MMP sequences and 9 OTU sequences obtained in this study). After multiple sequence alignment and manual refinement using Clustal W in IQ-TREE, the phylogenetic tree was constructed using the Neighbor-Joining method with 1000 bootstrap replicates and then visualized and refined using the iTOL online platform (https://itol.embl.de, accessed on 2 April 2025).

Phylogenetic tree was constructed using 88 known MMP 16S rRNA gene sequences retrieved from the NCBI database, along with the 9 representative OTU sequences obtained in this study, with *Candidatus* Magnetobacterium bavaricum (X71838) designated as the outgroup (Figure 6 and the detailed data are provided in Supplementary Material S1). The tree revealed two major clusters: 17 sequences grouped within the eMMP clade, and 64 sequences clustered within the sMMP clade.

The eMMP clade further bifurcated into two subclades. One subclade contained SHE6 (PV810352) and SHE7 (PV810356), which clustered with three sequences from the intertidal zone of Marseille, France (KY778006, MH013386, MH013388). Given that their similarity to the closest NCBI sequences was below 95%, these OTUs are inferred to represent a novel genus of eMMPs within the family Desulfobacteraceae.

The second eMMP subclade comprised SHE1–SHE5 along with seven reference sequences from diverse locations: the Xisha coral reefs (KY921894), Marseille, France (KT722334, KT722335, MH013381, MH013390), Rongcheng Yuehu (KF925363), and Qingdao, China (HQ857738). All sequences in this subclade belong to the genus *Candidatus* Magnetananas within the Desulfobacteraceae family.

Within the sMMP clade, the putatively novel species SHS1 formed a distinct subclade with three sMMP sequences from Marseille, France (MH013383, MH013387, MH013385), all belonging to the genus *Candidatus* Magnetoglobus. Meanwhile, SHS2 clustered with three MMP strains from the Xisha coral reefs (KY921895, KY921896, KY921897), forming a subclade identified as *Candidatus* Magnetoglobus multicellularis [17]. All sMMP sequences also fall within the family Desulfobacteraceae.

## 4. Discussion

### 4.1. Magnetosome Characteristics of Multicellular Magnetotactic Prokaryotes

As signature organelles of magnetotactic bacteria, magnetosomes vary in size, composition, and morphology in response to environmental conditions, exhibiting distinct characteristics [49,50]. In this study, both eMMPs and sMMPs synthesized only bullet-shaped magnetosomes, yet significant differences were observed in their arrangement, quantity, and dimensions. The magnetosomes within eMMPs were more regularly arranged and significantly more numerous than those in sMMPs. Moreover, the bullet-shaped magnetosomes in eMMPs in this study were notably smaller in both length and width than those previously reported in other eMMPs (Table 2). These differences may be closely related to their biological functions and magnetosome biomineralization mechanisms.

Notably, as early as 1993, Bazylinski et al. [51] detected the presence of copper in greigite (Fe_3_S_4_) magnetosomes from MMPs samples collected in Morro Bay, California. The copper content accounted for approximately 0.1–10 at% of iron, and Scanning-transmission electron micrograph of magnetosomes revealed that the copper signal coincided with the magnetosomes. This suggests that copper likely exists as surface-adsorbed species or discrete copper sulfide nanoparticles, potentially associated with a cellular detoxification mechanism. It is worth emphasizing that this study is the first to report the presence of copper (Cu) within Fe_3_O_4_ magnetosomes of sMMPs. HRTEM analysis showed three different cases of Cu detection. First, in eMMPs, only a very weak signal-to-noise image was seen across the whole field of view (Figure 4d3), which likely represents background Cu. Second, the strongest Cu signal appeared in sMMPs, where Cu was evenly distributed throughout the entire crystal (Figure 4i3). Third, an intermediate Cu signal was found in sMMPs, where Cu appeared together with the crystals (Figure 4m3). To measure the elemental composition, EDXS was used on eMMPs (Figure 4b) and sMMPs (Figure 4g,k), focusing on the atomic percentages (atom%) of O, Fe, and Cu, as well as their ratios (Table 3). The results showed that eMMP magnetosomes contained much less Cu, with lower (Fe + Cu)/O and Cu/Fe ratios compared to sMMP magnetosomes. Elemental mapping confirmed this: almost no Cu signal was detected in eMMP magnetosomes (Figure 4d3), while clear Cu signals were found in sMMP magnetosomes (Figure 4i3,m3). EDXS peak analysis gave further support. In eMMP magnetosomes, the Cu peak was almost the same as the background signal from the cell (Figure 4e). In contrast, in sMMP magnetosomes, the Cu peak was much stronger than the background (Figure 4j,n), indicating the presence of Cu in the magnetosomes. However, since sMMP contain a relatively high Cu background, part of the detected Cu signal may originate from the background rather than the magnetosomes alone. At this stage, it is still unknown whether Cu in magnetosomes depends on the species or on the strain.

Furthermore, Pósfai et al. [52] compared the elemental composition of magnetosomes from magnetotactic bacteria across different geographical locations and found that copper enrichment exhibits notable species- and site-specificity. This phenomenon was only observed in samples from specific regions, such as the sMMP sampling site in Morro Bay, CA, USA. The researchers hypothesized that copper may not be incorporated into the magnetosome crystal lattice via doping but rather exists as surface-adsorbed species or discrete copper sulfide nanoparticles. In contrast, Ikeda et al. [53] demonstrated that the influence of Cu on the artificially synthesized nanostructure of Fe_3_O_4_ particles was investigated. X-ray absorption fine structure analysis showed that Cu is substituted for octahedral Fe and exists as Cu_x_Fe_(3−x)_O_4_ in Fe_3_O_4_. The first-principle calculation showed that lattice strain resulted around the substituted Cu. This suggests that this lattice strain around substituted Cu inhibited the lattice growth and contributed to the refinement of Fe_3_O_4_ particles. In the present study, as shown in Figure 4h, Cu incorporation did not alter the lattice spacing of Fe_3_O_4_. However, in Figure 4l, the measured lattice spacing of one magnetosome was d = 4.51 Å, closest to the d-spacing of the {111} planes of Fe_3_O_4_ (d = 4.85 Å). Variations in copper atomic percentages were observed among different magnetosomes within the same cell and at different locations within individual magnetosomes, indicating heterogeneous distribution of copper. It is thus speculated that Cu may either be adsorbed on the surface of magnetosomes or sporadically present within Fe_3_O_4_ as Cu_x_Fe_(3−x)_O_4_, partially substituting for Fe. This substitution may introduce thermodynamic instability during crystal growth, thereby inhibiting further lattice development.

Based on these findings, we hypothesize that: (1) Cu incorporation may be specific to sMMPs and influenced by geographic location; (2) Cu deposition in sMMP magnetosomes may be a sporadic event, potentially triggered by specific exposure to Cu; (3) the use of copper grids for TEM observation without elemental mapping may have led to underestimation of Cu presence in magnetosomes in previous studies.

**Table 2:** Comparison of the characteristics of different types of MMPs.

Type	Sampling Site	Size (µm)	Magnetosome				Ecological Environment	NCBI Accepted Sequences	NCBI Accepted Sequences	Reference
			Type	Shape	Composition	Size (nm)				
**eMMPs**	**Sanya, China**	**(9.74 ± 1.52) µm × (8.15 ± 1.54) µm**	**I**	**Bullet-shaped**	**Fe_3_O_4_**	**90.1 ± 22.8 (L), 34.0 ± 4.9 (W)**	**Intertidal zone**	**Tropical Monsoon Climate**	**PV810351–PV810352, PV810354–PV810356, PV810358–PV810359**	**This study**
	Qingdao, China	(9.6 ± 1.2) μm × (7.8 ± 0.9) μm	I	Bullet-shaped	Fe_3_O_4_	102 ± 24 (L), 38 ± 6 (W)	Intertidal zone	Temperate Monsoon Climate	HQ857738 (*Candidatus* Magnetananas tsingtaoensis)	[12]
	Rongcheng, China	(9.18 ± 1.01) μm × (7.41 ± 0.76) μm	I	Bullet-shaped	Fe_3_O_4_	115 ± 27 (L), 39 ± 5 (W)	Intertidal zone	Temperate Monsoon Climate	KF925363 *(Candidatus* Magnetananas rongchenensis)	[14]
			II	Bullet-shaped Irregularly shaped	Fe_3_O_4_	—				
					Fe_3_S_4_	102 ± 14 (L), 78 ± 13 (W)				
	Marseille, France	(8.1 ± 1.2) μm × (6.5 ± 1.1) μm	I	Bullet-shaped	Fe_3_O_4_	119 ± 29 (L), 40 ± 4 (W)	Sediment	Mediterranean Climate	KT722334 (*Candidatus* Magnetananas sp. SF-1)	[37]
	Drummond Island, China	(10.3 ± 1.4) μm × (8.2 ± 1.2) μm	I	Bullet-shaped	Fe_3_O_4_	115 ± 24 (L), 44 ± 6 (W)	Sediment	Tropical Monsoon Climate	KT722335 (*Candidatus* Magnetananas drummondensis)	[37]
	Marseille, France	(6.93 ± 1.58) μm × (5.53 1.29) μm	—	—	—	—	Sediment	Mediterranean Climate	KY778001–KY778006, MH013381, MH013386, MH013388-MH013390	[16]
	Xisha Islands, China	(7.47 ± 1.6) µm × (6.04 ± 1.21) µm	I	Bullet-shaped	Fe_3_O_4_	134 ± 23 (L), 40 ± 4 (W)	Coral Reef	Tropical Monsoon Climate	KY921894	[15]
			III	octahedral	Fe_3_O_4_	40				
	Yitong Ansha Reef, China	—	—	—	—	—	Coral Reef	Tropical Monsoon Climate	MW929199	—
sMMPs	**Sanya, China**	**(5.64 ± 0.8) μm**	**I**	**Bullet-shaped**	**Fe_3_O_4_**	**97.2 ± 21.4 (L), 36.3 ± 3.4 (W)**	**Intertidal zone**	**Tropical Monsoon Climate**	**PV810353, V810357**	**This study**
	Little Sippewissett Salt Marsh, USA	(6.54 ± 0.93) μm	—	—	—	—	salt marsh	Temperate Oceanic Climate	DQ630668–DQ630712	[36]
	Araruama Lagoon, Brazil	(6.0–9.5) μm	III	Irregularly shaped	Fe_3_S_4_	88 (L), 71 (W)	salt marsh	Tropical Marine Climate	EF014726 (*Candidatus* Magnetoglobus multicellularis)	[17]
	Wadden Sea, Germany	5.7 μm	I	Bullet-shaped	Fe_3_S_4_	90 ± 21 (L), 40 ± 6 (W)	sandy sediments	Temperate Oceanic Climate	EU717681 (*Candidatus* Magnetomorum littorale)	[24]
	Pyramid Lake, USA	—	—	—	—	—	Sediment	Subtropical Arid Climate	GU784824	—
	Qingdao, China	(5.5 ± 0.8) μm	I	Bullet-shaped	Fe_3_O_4_	92 ± 20 (L), 35 ± 4 (W)	Intertidal zone	Temperate Monsoon Climate	HQ857737 (*Candidatus* Magnetomorum tsingtaoroseum)	[30]
	Rongcheng, China	(5.6 ± 0.9) μm	II	Bullet-shaped	Fe_3_O_4_	80.1 ± 16.1 (L), 33.6 ± 3.5 (W)	Intertidal zone	Temperate Monsoon Climate	KF498702 (*Candidatus* Magnetomorum rongchengroseum)	[19]
				Irregularly shaped	Fe_3_S_4_	63.9 ± 9.3 (L), 52.5 ± 7.5 (W)				
	Xisha Islands, China	(5.87 ± 1.37) μm	I	Bullet-shaped	Fe_3_O_4_	139.4 ± 36.3 (L), 39.2 ± 3.5 (W)	Coral Reef	Tropical Monsoon Climate	KY921895–KY921899	[15]
	Little Sippewissett Salt Marsh, USA	(4.33 ± 0.20) μm	—	—	Fe_3_S_4_/FeS_2_	—	salt marsh	Temperate Oceanic Climate	L06457	[54]
	Marseille, France	—	—	—	—	—	Sediment	Mediterranean Climate	MH013382, MH013383, MH013385, MH013387	—
	Sanya, China	(4.6 ± 0.2) μm	III	Irregularly shaped	Fe_3_S_4_	77 ± 11	mangrove	Tropical Monsoon Climate	MW356768	[40]
			II	Irregularly shaped Bullet-shaped	Fe_3_S_4_	80 ± 19				
					Fe_3_O_4_	88 ± 19 (L), 34 ± 5 (W)				
			I	Bullet-shaped	Fe_3_O_4_	78 ± 18 (L), 34 ± 4 (W)				
	Jinsha Bay, China	(4.78 ± 0.6) μm	I	Bullet-shaped	Fe_3_O_4_	87.0 ± 20.3 (L), 35.2 ± 3.5 (W)	Intertidal zone	Subtropical Monsoon Climate	ON007023 (*Candidatus* Magnetoradiorum zhanjiangense XL-1)	[20]
			III	Irregularly shaped	Fe_3_S_4_	72.8 ± 8.7 (L), 55.2 ± 7.3 (W)				
nMMPs	Pyramid Lake, USA	(7.5 ± 1.0) μm	—	—	—	—	Sediment	Subtropical Arid Climate	GU732821–GU732827	[21]
—	Marseille, France	—	—	—	—	—	Sediment	Mediterranean Climate	MH013384, MH013391	—

In the table, the bold entries indicate data obtained from this study. The magnetosome types are defined as follows: Type “I” refers to individuals that only produce bullet-shaped magnetosomes; Type “II” refers to individuals that produce both bullet-shaped and irregular-shaped magnetosomes; Type “III” refers to individuals that only produce irregular-shaped magnetosomes. The symbol “—” indicates that the information is unpublished or not included in the article.

Magnetosome size, morphology, and composition are strictly regulated by the magnetosome gene clusters (MGC), conferring species-specific traits [3,20,54,55,56], while also being influenced by environmental factors [50,57,58,59,60]. The distinct Cu uptake and accumulation behaviors between eMMPs and sMMPs may reflect differences in physiological needs, environmental adaptation, and magnetosome synthesis and function. Future studies should focus on: (1) elucidating the precise distribution and chemical state of Cu within magnetosomes; (2) investigating the influence of Cu on the biomineralization process; (3) examining the relationship between Cu incorporation and environmental adaptation in magnetosomes. Such research will advance our understanding of magnetosome biomineralization mechanisms and their ecological functions.

### 4.2. Distribution Patterns of Multicellular Magnetotactic Prokaryotes

Phylogenetic analysis in this study revealed the presence of potential new species within both eMMPs and sMMPs, providing new perspectives on the diversity and distribution of MMPs across environments. These putative novel species may possess unique physiological or ecological traits, offering insights into their environmental roles. We compared the ecological environments and climate types of all known MMP source locations (Table 2). Statistically, eMMPs show a relatively confined global distribution, predominantly found in Marseille, France—a region characterized by a Mediterranean climate—accounting for 88.2% of known eMMP sites. In contrast, sMMPs are distributed across a wider range of climate types, suggesting greater ecological adaptability and capacity to thrive under varied climatic conditions, consistent with existing reports [37].

We speculate that certain environments may possess features that favor the survival and competitiveness of sMMPs, or impose constraints that limit eMMPs, such as high salinity in salt marshes, hypersaline lagoons with high biomass and organic input, or unique mangrove marine habitats. In contrast, regions where both eMMPs and sMMPs coexist in nearly equal proportions, such as the Xisha Islands and Sanya (tropical monsoon climate), as well as Qingdao and Rongcheng (temperate monsoon climate) may provide relatively balanced ecological conditions that support the coexistence of both morphotypes. These areas share common features including high annual precipitation, suitable summer water temperatures, abundant nutrient inputs, stable seawater salinity, and sufficient sunlight, all conducive to maintaining metabolic activities and niche differentiation of diverse MMPs. Moreover, strong seasonal variations in these climates may offer complementary ecological niches for eMMPs and sMMPs, allowing them to alternate dominance in different seasons or microenvironments and achieve long-term coexistence—a pattern consistent with our observations.

Research on MMP diversity enhances our understanding of their presence in global tropical monsoon climates. This work provides new resources for exploring MMP ecological adaptation across climate zones and facilitates further biogeographic and ecological research on MMPs in varied environments. Furtherly, enhance our understanding of microbial functional communities in tropical ecosystem.

## 5. Conclusions

This study analyzed the diversity of MMPs in sediments from Haitang Bay, Sanya, a region notable for its unique ecological resources and geographical setting. The results indicate the co-occurrence of both eMMPs and sMMPs morphotypes in the area. Using TEM coupled with EDXS, we observed that both eMMPs and sMMPs synthesized bullet-shaped magnetite (Fe_3_O_4_) magnetosomes. Notably, Cu was detected homogenously across the entire crystal body within magnetosomes of sMMPs, representing the first report of Cu incorporation into Fe_3_O_4_ magnetosomes. Analysis of 16S rRNA gene sequence revealed a high diversity of MMPs, comprising a total of nine OTUs, including three putatively novel species. Phylogenetically, all sequences belonged to the Desulfobacteraceae family within the Thermodesulfobacteriota phylum. Among these, two OTUs are proposed to represent two novel species within a new genus and one is identified as a novel species within the genus *Candidatus* Magnetananas. These findings significantly expand the reference sequence database for MMPs, particularly for eMMPs. Furthermore, by correlating the geographic and climatic features of all known MMP habitats, we suggest that eMMPs may prefer environmentally stable and nutrient-rich regions, whereas sMMPs appear to exhibit broader environmental adaptability. This insight provides new clues for understanding the ecological distribution and adaptive strategies of MMPs.

## Figures and Tables

**Table 1:** Closest sequences in GenBank to representative OTU sequences of MMPs from Haitang Bay in Sanya city.

Type	OTUs	Number of Sequences	Most Similar MMPs Sequences (Accession Numbers)	Similarity
eMMPs	SHE1	8	uncultured delta proteobacterium clone MMP PI3B-7 (KY921894)	99.28%
	SHE2	7	uncultured delta proteobacterium clone MMP PI3B-7 (KY921894)	98.82%
	SHE3	2	*Candidatus* Magnetananas rongchenensis (KF925363)	99.15%
	SHE4	2	*Candidatus* Magnetananas rongchenensis (KF925363)	99.41%
	SHE5	8	uncultured prokaryote clone SP-6 (MH013390)	99.28%
	SHE6	1	uncultured prokaryote R3-34 (MH013388)	94.50%
	SHE7	1	*Candidatus* Magnetananas sp. SF-1 (KT722334)	94.03%
sMMPs	SHS1	2	uncultured delta proteobacterium clone MMP PI7B-6 (KY921895)	96.99%
	SHS2	5	uncultured delta proteobacterium clone MMP PI7B-6 (KY921895)	99.54%

**Table 3:** The proportion (atom%) of O, Fe, Cu and their ratios in MMP magnetosomes.

Type	Point	O (%)	Fe (%)	Cu (%)	(Fe + Cu)/O	Cu/Fe	Cu/Fe (Averege)
eMMP	BC1	90.21	0.20	4.35	0.05	21.75	21.75
	MS1	84.41	7.70	3.47	0.13	0.45	0.27
	*	76.04	18.52	2.91	0.28	0.16	
	*	75.70	17.37	3.75	0.28	0.22	
	*	80.30	12.60	3.35	0.20	0.27	
sMMP	BC2	68.20	0.26	27.18	0.40	104.54	104.54
	MS2	62.77	22.71	11.62	0.55	0.51	0.38
	*	57.61	31.57	9.20	0.71	0.29	
	*	58.12	28.34	10.90	0.68	0.38	
	*	60.02	28.66	9.24	0.63	0.32	
	BC3	65.46	1.06	27.92	0.44	26.34	26.34
	MS3	54.41	33.55	9.85	0.80	0.29	0.34
	*	54.52	31.39	10.82	0.77	0.34	
	*	53.81	30.87	11.83	0.79	0.38	

BC indicates the blank control points in the non-magnetosome regions within the cells. MS refers to the points on the magnetosomes (MS1 corresponds to the EDXS of magnetosomes in Figure 4b of eMMPs, while MS2 and MS3 correspond to the EDXS of magnetosomes in Figure 4g and 4k of sMMPs, respectively). The symbol “*” indicates different points on the same magnetosome.

## Data Availability

Publicly available datasets were analyzed in this study. Additionally, all relevant genome sequences were obtained from GenBank, and the genome sequences acquired in this study (PV810351-PV810359) have been deposited into GenBank (https://blast.ncbi.nlm.nih.gov/Blast.cgi, accessed on 23 June 2025).

## Supplementary Materials

The following supporting information can be downloaded at: https://www.mdpi.com/article/10.3390/microorganisms13112624/s1, Figure S1: Sediment texture at the sampling sites. Video S1: motility of the MMPs. Supplementary Material S1: MMPs 16S rRNA gene sequences.

## Abbreviations

The following abbreviations are used in this manuscript:

MTB	Magnetotactic bacteria
GTDB	Genome Taxonomy Database
MMPs	multicellular magnetotactic prokaryotes
eMMPs	ellipsoidal multicellular magnetotactic prokaryotes
sMMPs	spherical multicellular magnetotactic prokaryotes
nMMPs	non-magnetosome-forming multicellular magnetotactic prokaryotes
WGA	whole-genome amplification
SEM	Scanning Electron Microscopy
PBS	Phosphate-buffered saline
TEM	Transmission Electron Microscopy
HRTEM	High-resolution Transmission Electron Microscopy
EDXS	energy-dispersive X-ray spectroscopy
XRD	X-ray diffraction
MDA	multiple displacement amplification
OTUs	Operational Taxonomic Units
MGC	magnetosome gene clusters
