# Supplementary figures and images for: Diversity of Multicellular Magnetotactic Prokaryotes in Sanya Haitang Bay
Source: Microorganisms. 2025 Nov 19;13(11):2624. doi: 10.3390/microorganisms13112624 (PMC12654384; doi:10.3390/microorganisms13112624)

**Figure S1.** Sediment texture at the sampling sites

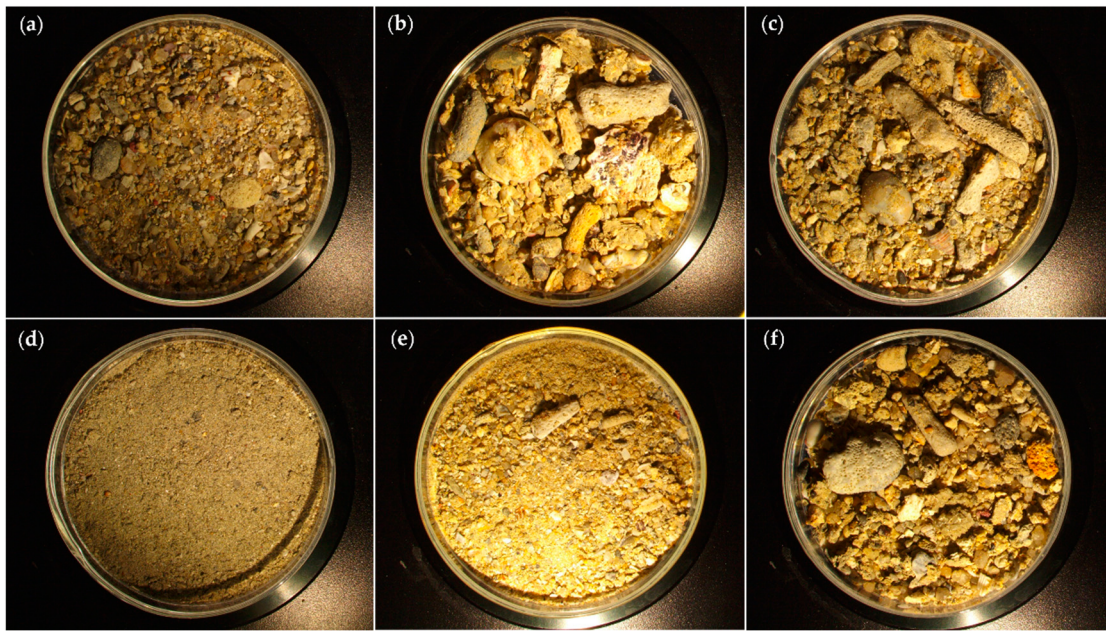

Supplement: Supplementary file 1 [file microorganisms-13-02624-s001.zip › Figure S1. Sediment texture at the sampling sites.pdf]
